# An unconventional uptake rate objective function approach enhances applicability of genome-scale models for mammalian cells

**DOI:** 10.1038/s41540-019-0103-6

**Published:** 2019-07-23

**Authors:** Yiqun Chen, Brian O. McConnell, Venkata Gayatri Dhara, Harnish Mukesh Naik, Chien-Ting Li, Maciek R. Antoniewicz, Michael J. Betenbaugh

**Affiliations:** 1 Department of Chemical and Biomolecular Engineering, Johns Hopkins University, 3400 North Charles Street, Baltimore, MD 21218 USA; 2 Department of Chemical and Biomolecular Engineering, Metabolic Engineering and Systems Biology Laboratory, University of Delaware, 150 Academy St, Newark, DE 19716 USA

**Keywords:** Biochemical networks, Computer modelling, Systems analysis

## Abstract

Constraint-based modeling has been applied to analyze metabolism of numerous organisms via flux balance analysis and genome-scale metabolic models, including mammalian cells such as the Chinese hamster ovary (CHO) cells—the principal cell factory platform for therapeutic protein production. Unfortunately, the application of genome-scale model methodologies using the conventional biomass objective function is challenged by the presence of overly-restrictive constraints, including essential amino acid exchange fluxes that can lead to improper predictions of growth rates and intracellular flux distributions. In this study, these constraints are found to be reliably predicted by an “essential nutrient minimization” approach. After modifying these constraints with the predicted minimal uptake values, a series of unconventional objective functions are applied to minimize each individual non-essential nutrient uptake rate, revealing useful insights about metabolic exchange rates and flows across different cell lines and culture conditions. This unconventional uptake-rate objective functions (UOFs) approach is able to distinguish metabolic differences between three distinct CHO cell lines (CHO-K1, -DG44, and -S) not directly observed using the conventional biomass growth maximization solutions. Further, a comparison of model predictions with experimental data from literature correctly correlates with the specific CHO-DG44-derived cell line used experimentally, and the corresponding dual prices provide fruitful information concerning coupling relationships between nutrients. The UOFs approach is likely to be particularly suited for mammalian cells and other complex organisms which contain multiple distinct essential nutrient inputs, and may offer enhanced applicability for characterizing cell metabolism and physiology as well as media optimization and biomanufacturing control.

## Systems biology: Unconventional modeling approach for predicting mammalian metabolism

Efficient application of genome-scale metabolic models for mammalian cells can be challenging due to the large number of restrictions posed on inputs including essential amino acid uptake rates. To overcome these limitations, a team led by Michael Betenbaugh at Johns Hopkins University estimated the essential amino acid inputs for Chinese hamster ovary (CHO) cells from readily measured growth rates. These predictions are then combined with the implementation of an unconventional approach for solving metabolic models based on minimization of uptake rates. Solutions predicted by this approach successfully distinguished metabolic differences between CHO cell lines that are not directly observable using conventional approaches and managed to identify the correct cell line from metabolic flux data. Implementation of this systems approach will enable users to better utilize genome-scale models of mammalian cells for improved understanding and control of biotechnology processes.

## Introduction

Constraint-based genome-scale models and flux balance analysis (FBA) have been widely used to investigate metabolic systems of various organisms. By connecting genotype to phenotype, genome-scale metabolic models (GeM) provide detailed information about biochemical reactions networks that compose cellular metabolism.^1^ Assuming pseudo-steady-state for the intracellular metabolites, one can limit the solution space of these underdetermined systems by optimizing one particular reaction via linear programming, referred to as the objective function. Besides a value of the optimized objective function, the resulting solution also provides information about intracellular fluxes within the system.^2^ A conventional objective function often used for biological entities is maximization of the growth rate, known as the biomass objective function (BOF), giving the underlying assumption that the “goal” of an organism is to maximize its reproduction, from a perspective of adaptive evolution.^3^ This optimization approach has been well-studied and validated for many prokaryotic organisms such as *Escherichia coli*^4,5^ and *B. subtilis*.^6,7^ However for a more complex metabolic system such as mammalian cells, fewer successful constraints-based and FBA-related models have been published.^8^ Chinese hamster ovary (CHO) cells represent the most widely used host cell for therapeutic recombinant protein production and have been gaining increased attention for in-silico modeling to better understand the metabolism. Prior to the development of CHO genome-scale models, constraint-based modeling work done on CHO cells included studies using metabolomics and FBA to investigate key metabolism such as energy consumption and lactate production,^9,10^ using a non-specific mammalian cell genome-scale model and *Mus musculus* metabolic model.

A recent CHO-specific genome-scale model contains information on 1766 genes, 6663 reactions, and has led to the generation of three cell line-specific models (CHO-S, CHO-K1, and CHO-DG44).^11^ The model was validated by predicting growth rates using sets of metabolic flux data from the literature, and was then applied to study the tradeoff between growth and recombinant protein production.^11^ The published CHO GeM has demonstrated its value in facilitating the studies of CHO metabolism. For example, one study tailors the generic CHO GeM into host and recombinant cell-specific models and integrated with multi-omics data to understand the differences of genotypic and phenotypic traits between wild-type and recombinant CHO cells.^12^ Another study perturbed the constraints of a CHO GeM to mimic the variation of medium composition and found that CHO cells stop growing when CHO-specific essential amino acids availability decreases, and limitations in non-essential amino acids can be overcome by enhancing amino acid biosynthesis reactions.^10^ Besides understanding intracellular physiology and predicting metabolic engineering consequences, constraint-based models can be useful for other applications such as model predictive control,^13–15^ for various organisms including yeast and mammalian cells.^16–19^

Computational models are useful tools typically because of their capability to describe multiple or hard-to-measure outputs from fewer or easy-to-measure inputs. However, current constraint-based modeling approaches do not fully exploit the value of detailed metabolic models, in particular for mammalian cells with complex, numerous and distinct nutrient inputs. Although intracellular fluxes can be estimated, required inputs are considerable and may be challenging to quantify rapidly. These represent some of the principal difficulties in extending constraint-based models to mammalian cell bioprocesses, eliciting the need for a modeling approach specifically tailored for mammalian cells.

In particular, mammalian cell growth predictions by FBA suffer from a significant complication due to the input requirement of numerous essential amino acids exchange fluxes as part of the constraints. Even slight disagreement between these uptake rate constraints and biomass composition in the model can result in optimization solutions being overly restrictive or even dictated by a single or a few potentially underestimated essential amino acid uptake rates. However, direct estimation of these essential amino acid uptake inputs is possible based on measured growth rates and a “essential nutrient minimization” (ENM) approach which solves for the absolute minimal consumption requirements. These rate estimations can be used to adjust the FBA constraints in order to resolve problematic mass balance constraints. Also, they can be transformed into real-time concentration predictions via a static optimization approach,^14^ which can be useful for bioreactor monitoring and control. In this study, we introduce an unorthodox FBA approach based on a set of uptake-rate objective functions (UOFs), which utilizes the measured growth rate as a constraint and independently minimizes the uptake rate of each individual non-essential nutrient. We demonstrate that the UOFs approach reveals insights concerning metabolic differences between mammalian cell line variants not evident from traditional BOF methodologies. Furthermore, sensitivity data derived from the UOFs solution provides a direct visualization of metabolic relationships between nutrients in the networks, providing enhanced characterizations of CHO physiology with applications ranging from cell line evaluation, metabolic engineering to media optimization and biomanufacturing control.

## Results

### Growth predictions of technical replicates reveal the impact of essential amino acid and rate-limiting fluxes

**Fig. 1:**
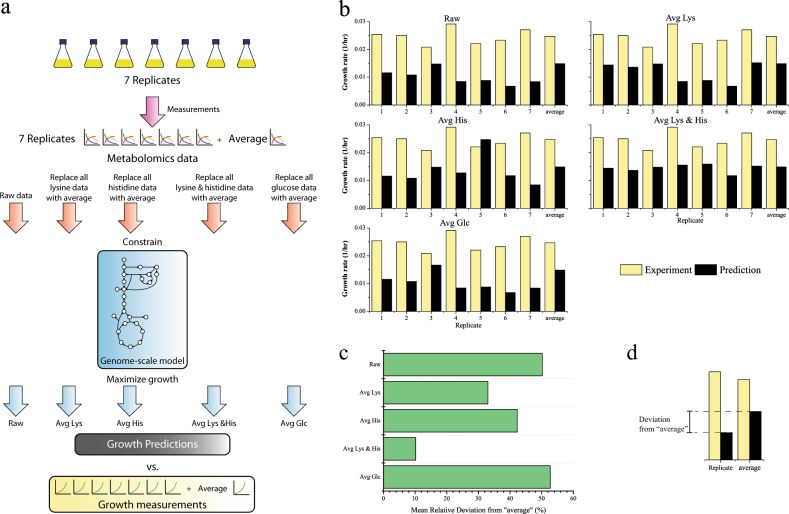
Effects of raw and averaged specific uptake constraints on growth rate predictions when using biomass objective function compared to experimental growth measurements for 7 replicates. **a** Schematic of different GeM growth predictions with raw or averaged data inputs of lysine, histidine, lysine plus histidine, and glucose; **b** growth rate measurements (yellow bars) versus growth rate predictions (black bars) before and after specific input averaging; **c** mean relative deviation of predicted grow rates using replicate 1–7 inputs compared to predicted growth rate using averaged inputs for all five raw/modified datasets; **d** deviation from “average” is defined as the difference between predicted growth rates using replicate and averaged inputs

We initially demonstrated how the presence of essential amino acids can exert a negative impact on the FBA of a mammalian cell model due to their values being rate-limiting factors for the conventional BOF, using a case study containing published CHO-K1 specific growth rates, 23 metabolites production and consumption rates for 7 cell culture replicates.^20^ Reported cell-density specific exchange rates of each replicate were converted to dry-weight specific rates assuming cell dry weight equaling 216.1 pg/cell.^11^ The FBA-predicted growth rates using metabolomics data of all the seven replicates as well as a prediction using all averaged flux data were compared against experimental measurements as illustrated in Fig. 1a. While all individual predictions for the individual raw inputs significantly underestimate the growth rate, the averaged flux inputs provide a higher growth rate value that more closely matches to the averaged measured value, indicating significant fluctuations for the individual measured fluxes within the 23 flux inputs (Fig. 1b). We, therefore, hypothesize that specific flux input values may be underestimated in some cases and as a result overly restrictive, lowering the predictions of the growth rate.

In order to elucidate which metabolites or fluxes limit the growth prediction for each individual replicate, we evaluated the dual price (also known as shadow price) of each metabolite for the BOF, which indicates how maximized growth rate changes in response to increasing the flux value of a particular metabolite under pseudo steady-state conditions. Shown in Supplementary Table 1 are the shadow prices for each of these metabolites for all replicates. In six of the seven replicates, only the dual prices of lysine and histidine are positive among the 23 flux inputs, suggesting these components represent the primary rate-limiting factors for growth maximization. The exception, replicate 3, indicates multiple rate-limiting factors exist. This is most likely due to energy being the rate-limiting factor in this particular FBA solution. However, the predicted growth rate of replicate 3 is closer to that of the averaged inputs, indicating that, unlike all other cases, replicate 3 is not significantly affected by deficiencies in the lysine and histidine input data.

To illustrate how predictions are biased by deviation in the lysine and histidine flux measurements, the growth rates were predicted with individual lysine and histidine flux constraints replaced by the replicate average. Averaged lysine or averaged histidine fluxes both result in increases of uptake flux constraints and growth rate predictions for multiple replicates (see Fig. 1b, Avg Lys or Avg His versus Raw), and averaging both (Avg Lys & His) brings most of the growth predictions to a similar and higher level, albeit lower than experimentally measured rates perhaps due to limitations with other constraints or inaccuracies in assumed cell dry weight. Shown in Fig. 1c is the mean relative deviation between the individual growth predictions for replicate inputs and the prediction using averaged inputs (definition shown in Fig. 1d), in which mean relative deviation drops from 50.2% for the Raw data to 10.2% for the Avg Lys & His, implying that lysine and histidine constraints are responsible for the interesting observation that averaged inputs yield the highest growth rate prediction. Averaging of glucose inputs had no impact on the growth rate prediction results except for replicate 3, in which the predicted growth rate increases by 13%. Indeed, these results agree with the dual prices with respect to BOF as illustrated in Supplementary Table 1, in which replicate 3 is limited by the energy sources such as glucose and the other replicates are limited, at least initially, by lysine or histidine. A distribution of measured lysine and histidine uptake rates (Supplementary Fig. 1) indicates that replicates 1, 2, and 7, whose growth predictions are limited by lysine, exhibit the lowest lysine uptake rates of the group; likewise for histidine-limited replicates 4, 5, and 6, their corresponding reported uptake rates are 50% less than the average. Thus 2 of the 23 flux constraints, which are both essential amino acids, dictate the upper limit of the growth maximization solutions for six of the seven replicates. These results illustrate that for mammalian modeling involving essential amino acids, underestimation of even a single exchange flux constraint can exert an outsized negative impact on the FBA-based growth rate predictions. Such a limitation can result in an inability to properly estimate the corresponding intracellular flux distributions. Unfortunately, typical analytical methodologies do not guarantee sufficient measurement accuracy that would prevent such flux underestimations.

### Essential amino acid consumption rates estimated by the “essential nutrient minimization” approach are comparable to the measured values

Given that essential amino acids can independently control the final FBA solution as essential elements of the BOF, it is possible that these amino acids can be rate-determining factors of cell growth. Since essential amino acid uptake rates may be directly related to growth, their consumption rate values can be estimated by finding the uptake requirements needed to sustain the observed growth rates, and one way to evaluate this approach is to compare measured amino acid uptake rates to model-predicted levels. Therefore, we began by revisiting the standard BOF and instead implementing a minimization approach in which the cells are assumed to incorporate nutrients at the minimal uptake rates with respect to the growth rate. This inversion of the objective function can provide a useful strategy to address the challenges in estimating the utilization rates for essential amino acids and other metabolites and eventually reduce the model inputs for FBA.

**Fig. 2:**
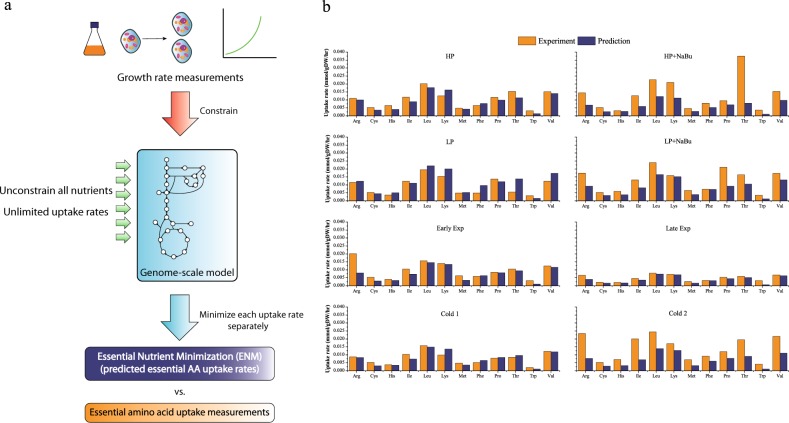
Approach to estimate essential amino acid consumption rates using growth rate data. **a** Schematic of Essential Nutrient Minimization (ENM) approach; **b** comparison of model-solved essential amino acid minimal uptake rates and measured exchange fluxes for 12 essential amino acids under different cultivation conditions. Literature experimental data include: High producer (HP), low producer (LP), sodium butyrate treatment (NaBu), early exponential phase (Early Exp), late exponential phase (Late Exp), and temperature shift (Cold 1 and Cold 2).^9,21,22^ Uptake rates are in units of millimole/gram dry weight/hour

First of all, one can solve for the “essential minimal” nutrient uptake rates for a particular growth rate after unbounding all nutrient uptake rates in order to permit unrestricted consumption of other nutrients while constraining growth and protein productivity, if available (Fig. 2a). The objective function is then set to minimize utilization of each specific substance. Here we define the term “essential minimum” as the minimal uptake requirement of a particular substance required to sustain a given growth rate and productivity regardless of how much other nutrients the cell can consume. This approach, called “essential nutrient minimization” (ENM) approach, can be used to identify model-specific essential nutrients as well as their absolute minimal uptake requirements corresponding to the observed growth rates. This ENM approach predicts non-zero fluxes for arginine, cysteine, histidine, isoleucine, leucine, lysine, methionine, phenylalanine, proline, threonine, tryptophan, and valine, which correspond to the 12 CHO-specific essential amino acids, as well as a limited glucose uptake rate. All non-essential amino acid uptake fluxes are zero when subjected to the same minimization approach due to reactions present in the model that allow synthesis of these nutrients. The ENM-solved essential amino acid uptake rates solved from experimentally measured growth rates are compared with measured exchange fluxes for three published studies and eight cultivation conditions.^9,21,22^ These growth and metabolomics datasets collected for various culture conditions were originally used to validate the CHO GeM.^11^ Shown in Fig. 2b is a comparison of essential amino acid uptake rates between the ENM solutions and measured values. For both high and low producers, early and late exponential phases, and one of the two cases in which cultures were subjected to temperature shift (Cold 1), the relative prediction error is 26% assuming measurement error is zero. This comparability between prediction and measurement across 12 different amino acids suggests that in unperturbed culture conditions, CHO cells consume essential amino acids at a rate near the theoretical minimum. This means that essential amino acids contribute mostly or only to synthesize biomass and recombinant proteins for CHO cells cultured in these conditions, and this may be due to a number of possible reasons. For example, it might be assumed that in many typical culture conditions, the enzymatic pathway efficiency of metabolizing the non-essential nutrients outcompetes the efficiency of degrading essential amino acid as energy sources, leading to CHO cell’s preference of consuming non-essential nutrients and only requisite amounts of essential amino acids. However, a noticeable underestimation of consumption rates can be observed for several essential amino acids in Fig. 2b when the cells were treated with sodium butyrate (HP + NaBu and LP + NaBu) and the second case in which cells were subjected to a temperature shift (Cold 2). A possible explanation for the larger average discrepancy in these cases is that culture perturbations led to a physiological response to environmental stress, causing the cells to consume more essential nutrients than the theoretical minimal growth requirement. In addition, tryptophan uptake rates are underestimated for the majority of the computational predictions. One possibility is that CHO cells consume and metabolize more tryptophan than minimal requirement. Another possibility is that tryptophan composition in the cell biomass experimentally is different from that in the model. Moreover, the results also help explain the unsuccessful BOF-based growth prediction in the low producer case of Fig. 2b, where the growth rate prediction exhibited a relative error as large as 60% with respect to the experimental data in the previous CHO GeM publication.^11^ Minimal amino acid flux predictions match closely to the experimental data except for phenylalanine and threonine, and among the two, the measured threonine uptake is much lower than prediction and has the same level of error as growth prediction, meaning that the growth prediction is directly dictated by the low measured threonine uptake flux for this particular case.

**Fig. 3:**
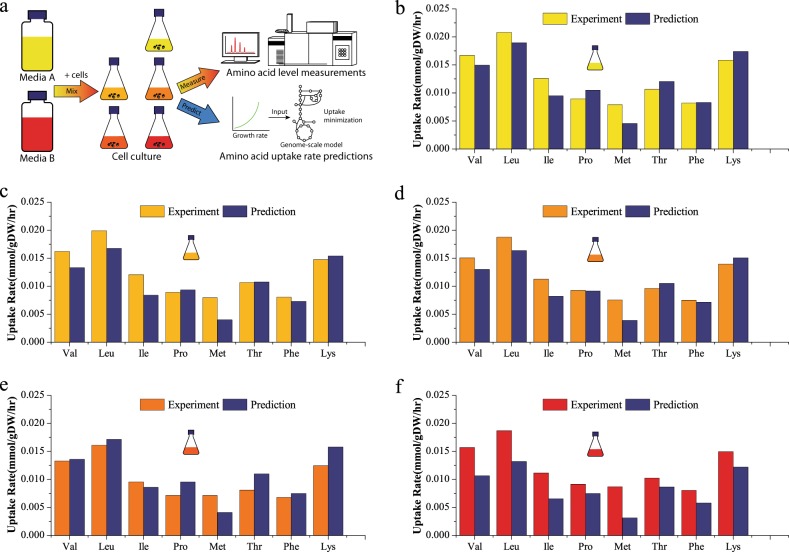
Comparison Of Model-predicted Minimal Essential amino acid uptake rates and measured exchange fluxes for an IgG-producing CHO-K1 cell line in various media blends during the early exponential phase. **a** Schematic of media-blend experimental measurements and model predictions; **b** 100% media A, **c** 75% media A + 25% media B, **d** 50% media A + 50% media B, **e** 25% media A + 75% media B, **f** 100% media B

To further examine if the predictable behavior of essential amino acids consumption can be observed in cultures having different media compositions, a batch experiment was performed with a suspension IgG1 producing CHO-K1 cell line grown in five mixtures of two different chemically defined media A and B (Fig. 3a). Viable cell density (VCD), glucose, 15 amino acids, and lactate concentrations were measured daily until all cells reached the death phase at ~167 h. Specific growth rates were estimated by fitting VCD data in the growth phase (0–94 h) to an exponential growth equation. All the cell dry-weight-specific consumption rates were calculated using concentration levels and integrated viable cell density from 18 to 94 h, assuming cell dry weight approximately equals to 216.1 pg/cell.^11^ Specific productivity was assumed unchanged to be the model’s “default number”, originally measured in another study.^9^ Since the flux magnitude through recombinant protein production is relatively small compared to the flux through biomass function, productivity does not have a significant impact on flux prediction results for this model. Flux predictions for the eight measured essential amino acids (arginine, cysteine, and tryptophan values were not available) obtained by this approach agree with the experimental values for all cases (Fig. 3b–f), and the relative magnitudes of each amino acid uptake rate are consistent between prediction and measurement and among the 5 different cases. However, methionine uptake is underestimated for all cases with a relative error as large as 64%, which may be due to methionine measurement error or biomass composition differences between the cell lines used in the experiment and that in the model. Obvious differences in culture behaviors can be observed for the 5 cases, confirming the impact of media composition on the cells. For example, cells cultured in 100% media B secreted glutamate at a higher rate than that in 100% media A. Despite obvious differences observed for cell behavior in the two media (details shown in Supplementary Data), measured essential uptake fluxes are conserved and consistent with our predictions. Moreover, no direct relation between essential amino acid concentrations and uptake rate was observed. For example, even though the measured initial proline concentration in 100% media A is more than 2 times higher than that in 100% media B, measured specific proline consumption rates demonstrate no significant difference for all 5 media blends. Thus for these CHO cells, it is reasonable to assume that uptake rates of the 12 essential amino acids are strongly related to growth and can be directly estimated by finding the minimal uptake requirement. Thus, one is able to avoid measuring 12 input constraints needed to conduct FBA.

### Unconventional uptake-rate objective functions (UOFs) for non-essential nutrients reveal metabolic differences across cell lines

**Fig. 4:**
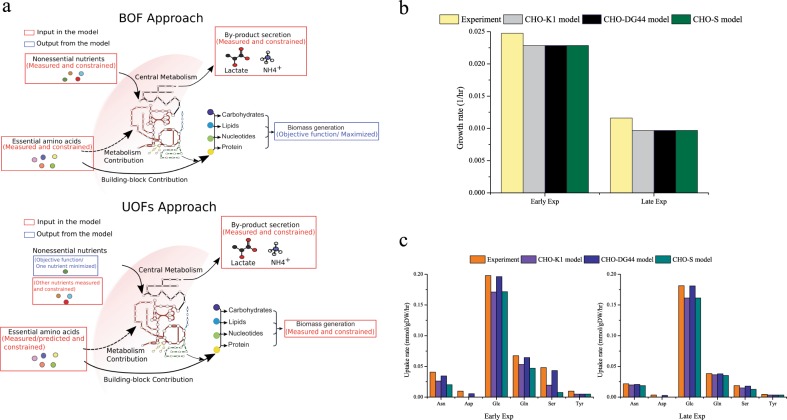
Demonstration of biomass objective function (BOF) and uptake-rate objective functions (UOFs)-based predictions versus experimental measurements for for CHO cells during early and late exponential phases. **a** Schematics of the BOF and UOFs inputs and outputs when applied to CHO genome-scale model; **b** growth rate predictions via BOF using three cell-line specific models and metabolomics datasets for fed-batch of CHO-DG44-derived cell line,^9^ for early and late exponential phases; **c** uptake rate predictions of glucose and 5 non-essential amino acids and measurements obtained via UOFs for these same datasets

Mentioned in the previous section, an “essential nutrient minimization approach” can be used to estimate the consumption rates of 12 CHO-specific essential amino acids directly from growth rate, and as a result, makes FBA more tractable for biotechnologists as they are able to measure fewer key metabolites in order to generate an intracellular flux distribution profile. Next, we applied an unconventional FBA approach that individually minimizes a number of uptake rates of non-essential nutrients, while the growth rate and all other available flux constraints are fixed (Fig. 4a). To demonstrate the merits of this UOFs approach, we first optimized the growth rates via BOF using three different CHO cell line models (CHO-K1, CHO-DG44, and CHO-S) for a CHO M250-9 fed batch culture^9^ (Early Exp and Late Exp cases in Fig. 2b). Shown in Fig. 4b, the BOF predictions present the correct experimental trends in decreasing growth rate with time for early and late exponential phases but interestingly no difference was observed in the three cell-line specific growth rate predictions.

We then applied the UOFs algorithm in which uptake rates of glucose and non-essential amino acids (only for those that are consumed by the cells) were minimized individually one at a time. Measured growth rates, IgG productivity and all measured metabolite exchange rates (except for the one to solve for) were set as constraints. Essential amino acid exchange inputs were corrected with the ENM-predicted uptake rates (as shown in Fig. 2) if the measured values were smaller. Interestingly, the FBA results revealed distinct differences among the three cell-line specific models (Fig. 4c). Simulation results of each nutrient indicated that the CHO-DG44, among the three cell line variants, requires more nutrients to sustain the given growth and flux constraints, indicating a more “resource demanding” metabolism in this experimental condition. Furthermore, for both early and late exponential phases, uptake rates predicted by the -DG44 model agree best with experimental measurements for all the substrates considered except tyrosine (in which all predictions were the same). Also noteworthy is that the -DG44 model correctly predicts consumption of aspartic acid while the other two models ignore aspartate consumption. Of note, CHO M250-9 is indeed a CHO-DG44 cell line producing an IgG product of commercial interest. These results demonstrate that the nutrient uptake behavior solved with the model of CHO-DG44, the cell-line used in these experiments, best describes the experimentally observed metabolic profile, and unlike the BOF-based results, successfully captures metabolic differences among the three cell lines. Although cell line differences might exist in the BOF-driven solution, they are not readily observable and likely hidden in the thousands of intracellular fluxes predicted. To demonstrate how the UOFs approach can impact the intracellular flux solutions, viable flux ranges of several intracellular carbohydrate metabolism reactions solved independently by the BOF and a single UOFs solution (glucose), are shown in Supplementary Fig. 4 for the early exponential case (see Fig. 4b, c). For the selected reactions in the BOF solution, CHO-K1 and CHO-S exhibit similar fluxes while CHO-DG44 has the most distinct flux profile compared to the other two cell lines. The fluxes obtained by the UOFs methods also varied, meaning that UOFs approach is available as a tool for evaluating flux profiles across cell lines and culture conditions. Interestingly, the resulted ranges of viable flux of UOFs solution are narrower than that of BOF, potentially due to the fact that the UOFs approach uses measured growth rate as a constraint. Alternatively, BOF’s predicted growth rate is lower than UOFs’ growth rate constraint, resulting in less contribution of nutrients to biomass and thus more available as “free fluxes” in the BOF solutions. Thus this UOFs mechanism can be a powerful method for analyzing metabolic systems that require multiple nutrient inputs, which typically exist in mammalian cell cultures.

### Dual prices of metabolites for uptake rates describe nutrient substitution and metabolic stress inflicted by excessive methionine

**Fig. 5:**
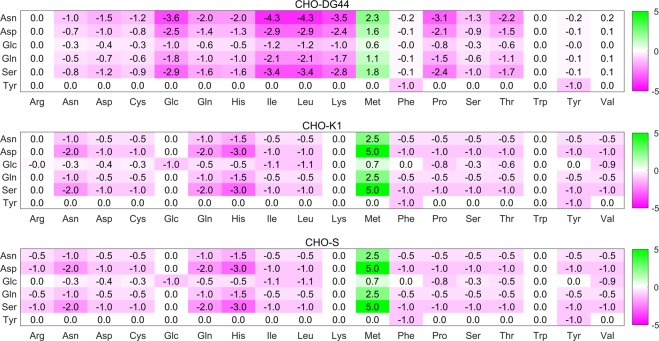
Dual price sensitivity of uptake fluxes for 6 non-essential nutrients in response to 17 nutrient exchange fluxes for CHO-DG44, -K1 and -S during early exponential phase. Positive and negative values represent increase or decrease, respectively, of uptake requirement in response to increasing consumption of amino acids and glucose. x-axis: Increase in 1 unit of specific amino acid or glucose availability; y-axis: Resulting increase/decrease of each nutrient uptake requirement

As mentioned previously, dual prices of metabolites can be calculated relative to BOF to predict the metabolite which upon increasing will potentially improve the growth. However, BOF-based dual prices provide very limited information, especially for the mammalian models since the dual price is zero unless the metabolite is rate-limiting for the FBA, and having 10–12 innate and potentially independent rate-limiting metabolites in the model will very likely result in having only one non-zero value for the optimized solution. Furthermore, the resulting dual price is not meaningful if rate-limiting flux is an artifact due to a slightly underestimated experimental exchange flux. On the other hand, dual prices calculated based on UOFs tell how many units of each uptake requirement will change if one increases one unit of another metabolite present in cell. Figure 5 shows dual prices of all the inlet metabolites with respect to each uptake flux from solutions in Fig. 4c for the three cell lines at the early exponential phase. Here each metabolite carries a distinct value presenting the relationship with each uptake flux. Since an IgG producing CHO-DG44 cell line (CHO M250-9) was used to generate the experimental data, results from CHO-DG44 model may contain the most relevant dual price values. Due to the use of measured flux profiles, these solutions do not represent a general analysis of the particular cell line but are scenario-specific for the particular experimental results used here, and thus it may not be appropriate to directly compare values between different solutions. Nevertheless, since every single dual price was calculated based on a corresponding optimized solution where each single uptake flux objective function was kept at its minimum, qualitative comparisons can provide knowledge about metabolic similarities and differences across the different cell lines. For example, for all three cell lines tyrosine can only be converted from phenylalanine at 1:1 ratio, as only the dual price entry for tyrosine (Tyr, y-axis) is phenylalanine (Phe, x-axis) and itself (Tyr, x-axis) at −1. Tryptophan is not directly related to any of the five metabolites considered here since the corresponding dual prices are zero. Also, for all three cell lines increasing methionine (Met, x-axis) will lead to elevated uptake requirement of five of the six nutrients excluding tyrosine. The reason is because the CHO cells require extra energy and other metabolites such as serine to catabolize methionine. The result also implies that rather than relieving other nutrient demands, excessive methionine uptake inflicts a metabolic burden on the cells. Arginine in CHO-S is metabolically connected to asparagine, aspartic acid, glutamine and serine, unlike in the other two cell lines which indicate negligible conversion from arginine (Arg, x-axis). Similarly, increasing valine consumption (Val, x-axis) exerts a very slight metabolic demand on other nutrients for the CHO-DG44 solution as indicated by the slight positive dual prices, while no such demand is evident in CHO-K1 and CHO-S dual prices of valine. These results reflect metabolic differences between CHO-DG44 and the other two cell lines, indicating fewer interrelated amino acid metabolic reactions available in the CHO-DG44 model and may potentially explain, at least in part, why CHO-DG44 requires slightly more nutrients than the other two cell lines in the simulation given the same hypothetical flux exchange profile.

## Discussion

The widely used BOF maximization approach has inherent limitations when applied to mammalian cells due to their complex nutrient requirements, including essential nutrients that the mammalian cells are not capable of synthesizing. Liebig’s law of the minimum states that agricultural yield is determined by the availability of scarcest essential nutrient resource.^23^ In the case of constraint-based models, this “Liebig’s barrel effect” also exists that the optimized maximal biomass reproduction rate is dictated by one or more rate-determining nutrient uptake rates. Therefore, from the perspective of FBA, each and every individual resource must be sufficient to satisfy the material balance for biomass generation in order to achieve the observed rate. Indeed, such mass balance violations have even been observed when modeling the prokaryotic *Escherichia coli*, and as a result the biomass composition was modified accordingly.^24^ These nutrient uptake restrictions detract from the reliability of a BOF-based mammalian cell FBA, because underestimation of even one amino acid consumption rate may significantly bias the linear program solution. As an attempt to address this limitation, one can identify and reformulate poor datasets by searching for flux constraints that make the material balance infeasible. For example, Hefzi et. al. ^11^ performed flux variability analysis to find the range of each exchange reaction that results in a feasible FBA solution. However, despite the wide range of feasible values, some upper limits of essential amino acid exchange fluxes were still slightly lower than the values needed to be consistent with observed growth rates, and as a result, the predicted growth rates were reduced. Considering how the disagreement between uptake rates and biomass composition data of essential amino acids can impact FBA results for mammalian models, it is recommended to pay extra attention to critical measurement values and appropriate corrections should be applied before using them to perform FBA.

Alternatively, the approach taken in this study was to directly estimate those essential amino acid uptake fluxes by solving for the “essential minimum” consumption requirements based on growth measurements. The predicted uptake rates are comparable with the experimentally measured values for a variety of unperturbed culture conditions (Fig. 2). As a result, researchers may reasonably avoid the effort-consuming analytical approaches required to obtain the information of consumption rates for each essential amino acid. However, the predictions demonstrated are based on several key assumptions. First of all, we assume that the model includes a reliable biomass composition of CHO cells. While the information of amino acid compositions in proteins were obtained from five different CHO cell lines,^9^ the total biomass composition used in BOF are not CHO-specific^11^ and thus may affect the accuracy of essential amino acid predictions based on growth rates. Second, we assumed a consistent cell dry weight of 216.1 picograms per cell for all cell lines based on the biomass composition used in the CHO genome-scale models.^11^ Other studies have reported various cell dry weight can range from about 300 pg/cell to as large as 770 pg/cell,^22,25–27^ and uncertainties in cell dry weight can impact the estimation of dry-weight-specific uptake rates. Finally, we assumed that the biomass composition and cell weight remains unchanged during 18–94 h culture period, which may not be realistic in all cases. Thus a more comprehensive and accurate set of information about CHO-line-specific biomass composition and dry weight is desired for even more reliable amino acid uptake rate predictions.

Nonetheless, this rate prediction approach can refine existing problematic constraints for FBA by preventing mass balance conflicts between constrained growth rates and essential nutrient exchange fluxes. This enables the implementation of the alternative UOFs objective function described here involving minimization of the uptake rate of non-essential nutrients. Previous studies have examined different objective functions that are potential candidates for representing the metabolic goal of organisms and considered selection of minimizing substrate utilization. Knorr, Jain, and Srivastava^28^ studied the selection of the most appropriate objective function using an *Escherichia coli* genome-scale model and stated that minimization of succinate does not predict the experimentally measured values. Savinell and Palsson^29^ investigated the effect of minimizing ATP production, NADH production, moles and mass of nutrient uptake for but observed that minimization of total nutrient uptake was not appropriate for hybridoma cells. Indeed, in a nutrient-rich environment, one might claim that the goal of an organism is not to minimize nutrient consumption. However, the validity of this nutrient minimization approach is actually supported for a variety of reasons. First of all, the UOFs approach has a theoretically equivalent underlying assumption as the BOF, where the BOF assumes organisms use their given nutrient availability to maximize growth, and the UOFs states that the organisms’ nutrient uptake is the minimal amount to sustain a given growth rate. BOF approach has been proved to work for a number of organisms including CHO according to the publication of CHO genome-scale model, thus the UOFs theoretically should generate similarly accurate predictions. Also, from the perspective of mass balances, the possible solutions will converge as the degrees of freedom decrease, thus the minimization approach will describe the actual behavior if enough relevant constraints are applied. Thus, although it may not be appropriate to apply the UOFs approach if the organism is known to systematically consume more nutrients than its metabolic requirement, this approach will be valid and even preferable if enough relevant constraints are available (e.g., enhanced lactate secretion fluxes may address excessive glucose consumption fluxes) and furthermore provides enhanced sensitivity in predicting insights into metabolic variability as described below.

**Fig. 6:**
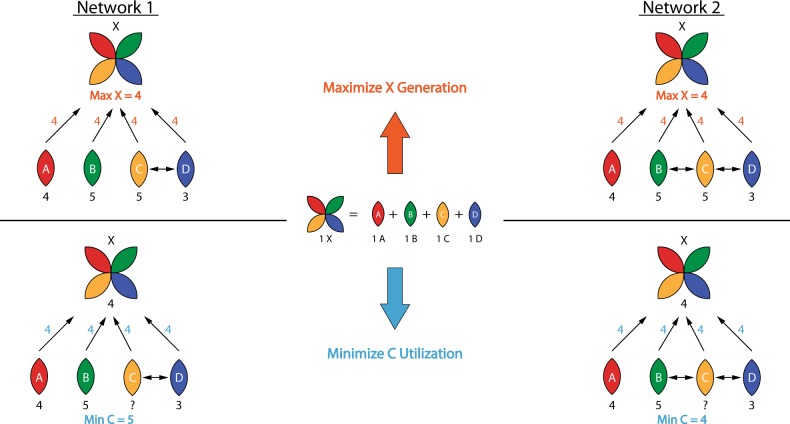
Model FBA problem containing 2 alternative hypothetical networks. Comparison between maximizing X (upper) and minimizing component C (lower) illustrates the enhanced sensitivity of the minimization approach for certain networks

Indeed, for a network including “macro-reactions” such as biomass generation which requires a combination of multiple elements such as energy sources and building blocks for protein, minimizing individual elements is distinct from maximizing biomass generation and is more capable of demonstrating metabolic differences between networks. This minimization approach takes into account the availability and excess of each single element and the metabolic connections among them, while classic maximization solutions may ignore some of these. As a simple example, FBA problems for 2 different hypothetical networks shown in Fig. 6 illustrate the difference between the two approaches when applied to models such as those present for mammalian cells. For the two hypothetical networks, a macro-component X (analogous to biomass protein) is composed of 1 unit of each individual building block components A, B, C, and D (analogous to amino acids), and Network 2 contains one more reaction than Network 1 (inter-conversion between B and C). With the given constraint on the availability of four components, maximizing production of X gives the same answer 4 for both networks, due to the fact that component A is rate-limiting (analogous to an essential amino acid). However, when the generation of X is constrained to 4 units and instead minimizing the utilization of C is the objective function, the two solutions vary and Network 2 yields a smaller minimized value. This difference in metabolic capabilities explains the observations in Fig. 4c in which two different cell lines yielded different solutions for the UOFs case while the two cell lines had equivalent solutions for the BOF case. In cases where multiple rate-limiting components are present, the UOFs are able to reveal a greater amount of information concerning metabolic differences, specifically, metabolic “flexibility”.

Such a difference indicates the UOFs to be a more suitable choice for selecting cell lines or clones by providing information about nutrient consumption efficiency and, potentially, viability in specific media environments. From the perspective of intracellular flux analysis, the FBA-derived flux profile is likely to be reliable only if the optimized objective function is consistent with the experimental data. Compared to BOF, linear program solutions based on UOFs are more sensitive to “system-level” variations, not only because UOFs calculate every single uptake flux, but also due to the reason that the BOF optimization does not consider other additional fluxes that do not contribute or detract from to the optimal solution of biomass generation (e.g., unconstrained by-product secretion). Thus an accurate prediction by UOFs may be more reliable for intracellular flux analysis than one obtained from a BOF. This advantage of being more sensitive increases the capability of identifying cell line variants from metabolomic flux data, leading to increased potential to obtain physiological or genomic insights from metabolomics data. However, the selection between BOF and UOFs approach is a trade-off between sensitivity and flexibility, where the UOFs may strictly necessitate certain measurement inputs as constraints which may not be required by the BOF to generate a solution. Moreover, the fact that UOFs uses growth rate as a constraint enables a robust analysis of intracellular flux profiles when the realistic cell growth rates can be reliably measured. The biomass generation reaction (growth rate) is the most important factor in FBA models which often drives the intracellular flux distribution since this reaction involves the greatest number of metabolites and its value largely determines the overall nutrient consumption and energy generation requirements. As mentioned before, BOF predictions may exhibit challenges in cases where nutrient constraints limit the range of possible growth solutions and hence lead to varied intracellular flux predictions. In contrast, the UOFs approach fixes growth rates as constraints; in cases where the uptake rate of a nutrient is inaccurately predicted, the impact on the intracellular flux profile may be more restricted to fluxes within that particular nutrient and its derivatives. Thus, the UOFs method is likely to still generate reasonable flux profiles for many of the metabolites involved in cell growth as long as the growth estimates are reliable.

Dual prices provide information on how the objective can increase/decrease relative to the change in a specific factor. In FBA, dual prices are commonly used to analyze the sensitivity of growth in response to a metabolite pool change and identify growth-limiting factors.^30^ This sensitivity data will also carry information regarding the energetics of metabolism.^31^ Dual prices calculated based on UOFs allow researchers to visualize the sensitivity of a metabolite uptake requirement with respect to changes in availability, providing useful information about metabolic relationships between metabolites such as inter-conversion, nutrient substitution as well as cost-benefit responses to metabolite pool perturbation. A previous study calculated the cost of synthesizing intermediates of glucose/glutamine consumption in hybridoma cells under different oxygen availability, by minimizing glucose/glutamine uptake rates after fixing glutamine/glucose exchange flux to minimum flux needed for biosynthesis and removing the catabolic pathways of essential amino acids.^32^ However, these sensitivity data were generated with artificial exchange constraints that will affect the optimization results and the optimization was not validated by experimental measurement, thus these values may not represent the actual metabolic cost of metabolites in hybridoma cells. Also, ignoring the catabolism of essential amino acids does not permit analysis of cases in which essential amino acids are over-consumed as shown in Fig. 2b. In the current study, our approach keeps the metabolic pathways intact and as a result incorporates constraints correction via a direct estimation of minimal essential amino acid uptake rates. The sensitivity results for CHO-DG44 shown in Fig. 5 are derived from validated FBA solutions in which the UOFs optimization results are consistent with experimental measurements.

The results in Fig. 5 illustrate the potential impact of perturbing the amino levels, such as negative consequences of increasing the intracellular methionine pool to a higher level. It suggests that if these cells use the resources in the most efficient way, they are likely to keep the methionine consumption to a minimum but sufficient rate to optimize its metabolism, which agrees with our general hypothesis made about estimating essential amino acid uptake rates. Also, it provides insights into strategies for media optimization in which it may be useful to limit methionine consumption during the cell culture process in order to minimize the potential undesired effects on the consumption of other nutrients. Indeed, a previous in vivo study has shown that excessive dietary methionine depletes serine in rat livers, and in turn obligates serine supplementation to reduce methionine toxicity,^33^ due to the fact that methionine catabolism in mammalian cells demands serine and ATP.^34^

As a result, this study introduces an alternative approach to predict the metabolic characteristics and performance for complex systems such as mammalian cells, for which conventional FBA approach are limited due to multiple overly restrictive or conflicting constraints. Prediction of essential amino acid consumption through the ENM approach prevents problematic rate-limitations by ensuring consistency between flux constraints and biomass composition, making our non-traditional UOFs approach feasible. The UOFs approach improves the capabilities and sensitivity of mammalian FBA with the underlying dual prices providing additional information about nutrient consumption, metabolic variations, and differences across cell lines. In addition, straightforward estimation of hard-to-measure amino acid concentration data from easy-to-measure cell counts and recombinant protein productivity may also enhance optimization of biomanufacturing (for example, see Supplementary Discussion). Indeed, predictions of essential amino acid consumption rates may be useful for facilitating monitoring of bioreactor operations and even model predictive control in the future.

## Methods

### Cell culture and quantification of extracellular metabolites

A CHO-K1 suspension cell line provided by National Institute of Health was used to perform amino acid consumption study. CHO cells were seeded at 0.3 × 10^6^ cells/mL and cultured in shaker flasks (Fisher Scientific) in incubators operating at 37 °C and constant 5% CO_2_ level. Direct mixture of two chemically defined media purchased from GE HealthCare Life Science and MilliporeSigma were used to generate different media compositions. 6 mM of glutamine was supplemented to the media before using. Culture aliquots were collected daily for analysis. Viable cell density was counted using hemocytometer and cell viability was estimated via trypan blue staining method. Glucose and lactate levels were measured using a YSI 2700 D select biochemistry analyzer (Yellow Spring Instruments). Concentrations of alanine, glycine, valine, leucine, isoleucine, proline, methionine, serine threonine, phenylalanine, aspartate, glutamate, lysine, glutamine, and tyrosine were measured using a GC-MS based method as described in a previous publication.^35^

### Growth and exchange rate calculation

Growth rates of CHO cells were assumed to follow exponential growth behavior:

1 $$N_x = N_{x,\,0} \cdot exp\left( {\mu \cdot t} \right)$$

Here *N*_*x*,0_ is the number of cells (counted in millions of cell) at initial time, *N*_*x*_ is the number of cells after culture time *t* (hour), and *μ* is the specific growth rate (1/hour). Specific growth rates were estimated by fitting the time and cell counts data to the exponential equation (1). Assuming CHO cells growing exponentially, the specific metabolite exchange rates were determined by dividing the metabolite concentration difference by the integration of cell density over time:^36^

2 $$r = \frac{{V \cdot \Delta C_i}}{{\mathop {\int }\nolimits_0^t N_xdt}} = \frac{{\mu \cdot V \cdot \Delta C_i}}{{N_{x,\,0} \cdot \left( {\exp \left( {\mu \cdot t} \right) - 1} \right)}}$$

Here specific exchange rate *r* has the unit of millimole per million cells per hour, *V* is the culture volume (mL), Δ*C*_*i*_ (mmol/L) is the concentration difference of metabolite *i* between two time points (initial time and after *t* hours). Specific exchange rates were converted to genome-scale model inputs, in the unit of millimole per gram dry weight per hour with an assumed cell dry weight.

### Constraint-based modeling

COBRA toolbox v3.0^37^ was used to conduct constraint-based modeling studies, running in MATLAB 2016b environment (The MathWorks Inc.). CHO global and cell-line specific genome-scale models (available from http://bigg.ucsd.edu/ and^11^) were used to perform analysis.

### Dual price analysis

In linear programs, dual price, also known as shadow price, is defined as the change in objective function as a result of a unit incremental change of a specific constraint.^30^ Such kind of dual problems study the marginal worth of a specific resource with respect to the optimization target. For FBA problems in this article, the dual price (λ_i_) of a specific uptake rate (*r*_*i*_) can be defined by the following formula:

3 $${\mathrm{\lambda }}_{\mathrm{i}} = \frac{{\partial Z}}{{\partial r_i}}$$

Here *Z* is the objective function for specific problems, which is, growth rate for BOF and non-essential nutrient uptake rate for UOFs. Dual prices in Fig. 5 and Supplementary Table 1 were solved using COBRA toolbox with Gurobi LP solver.

### Reporting summary

Further information on research design is available in the Nature Research Reporting Summary linked to this article.

## Supplementary information

Supplementary Information.

Supplementary Data.

Reporting Summary

## Data Availability

The authors declare that all data supporting the findings of this study are available within the paper and its supplementary information files. Additional data are available from the corresponding author upon reasonable request.

## Supplementary information

**Supplementary information** accompanies the paper on the *npj Systems Biology and Applications* website (10.1038/s41540-019-0103-6).
