# Supplementary material for: An unconventional uptake rate objective function approach enhances applicability of genome-scale models for mammalian cells
Source: NPJ Syst Biol Appl. 2019 Jul 23;5:25. doi: 10.1038/s41540-019-0103-6 (PMC6650483; doi:10.1038/s41540-019-0103-6)
Supplement: Supplementary file 1 — Supplementary Information. [file 41540_2019_103_MOESM1_ESM.pdf]

Supplementary Discussion

Dynamic ENM simulation accurately predicts real-time essential amino acid level throughout the batch process

To apply the model flux predictions to bioreactor predictive control, the first step is to turn flux estimations into concentration predictions. To achieve this as well as to prove that the ENM predictions are accurate for the entire batch process, a dynamic simulation was performed with the experimental dataset used in Figure 3 in order to generate a profile of predicted time-course essential amino acid levels. The static optimization approach was applied and steady-state solutions were solved iteratively for every time point and assumed to remain constant between adjacent time points. Daily viable cell density data was used to estimate exponential growth rate, and amino acid concentrations changes between two time points were calculated by multiplying the predicted cell-specific uptake fluxes by the integration of cell density over time. Starting from an initial amino acid concentration, daily concentrations were predicted according to measured VCD data and then compared to the experimental measurements (Supplementary Figure 2). For CHO cells cultured in 100% media A, this dynamic simulation predicts essential amino acid levels without any correction from measured amino acid concentration data, for the entire batch culture process up to 121 hours which includes a decline in growth. These accurate time-course simulations suggest this approach can be used to predict the dynamic changes in essential amino acid concentration levels in a CHO batch culture. A noticeable underestimation of valine, leucine and methionine consumption as well as the slight but unexpected proline concentration increase at the beginning of the process are exceptions. The former could be an indication of inaccurate biomass compositions whose error build-up is amplified by the simulation over time. The latter contradicts the model metabolic network since proline is considered to be an essential nutrient for CHO and typically is not synthesized endogenously and secreted. This apparent concentration rise could potentially be due to an evaporation effect or some other measurement method-related bias or, alternatively, limited synthesis and secretion of the amino acid. A proline concentration rise has not been detected in a number of previous

studies but two recent investigations did observe a bump up in proline levels using a similar quantification method.[1, 2]

This method represents a valuable tool to directly estimate real-time amino acid levels from viable cell density measurements. However, as mentioned, without correcting for measurement bias, such continuous simulations can lead to a significant build up in error during extended cell culture processes. It would be highly advantageous to utilize measurement data when available to track and correct the predictions. For bioprocesses involving routine amino acid quantifications using analytical methods, model estimation can be corrected by such data. We assume that the measurements are accessible as off-line data, and thus in the simulation for each time point we used data only from previous time points. In this scenario, one is able to use prior measurement and uptake flux data sets to update real-time concentrations. The predictions of current amino acid levels in Supplementary Figure 2 were then performed again after incorporating off-line data corrections for prior time points. With this correction applied, a better fit than purely simulative estimation can be clearly observed for the same data set (Supplementary Figure 3), as demonstrated by the fact that all the estimations match measurement values at the final time point with an average error 1.0%. This modification is especially beneficial when systematic bias is present. For example, the methionine uptake rate is generally underestimated in this particular study, as demonstrated in Figure 3, but this methionine consumption underestimation is significantly mitigated and the prediction error is reduced to 3.6% at 144 hour. Even if off-line data is not available at every time point, occasional correction will also be helpful by enabling the predictions to be adjusted to match measured levels whenever the correction is applied.

Supplementary Tables

Supplementary Table 1: Dual prices of metabolite with respect to biomass objective function

	Replicate							
	**1**	**2**	**3**	**4**	**5**	**6**	**7**	**Average**
**Ala**	0	0	0.1	0	0	0	0	0
**Arg**	0	0	0	0	0	0	0	0
**Asn**	0	0	0.1	0	0	0	0	0
**Asp**	0	0	0.1	0	0	0	0	0
**Cys**	0	0	0.1	0	0	0	0	0
**Glc**	0	0	0.2	0	0	0	0	0
**Gln**	0	0	0.1	0	0	0	0	0
**Glu**	0	0	0.1	0	0	0	0	0
**Gly**	0	0	0	0	0	0	0	0
**His**	0	0	0.1	8.2	8.2	8.2	0	0
**Ile**	0	0	0.2	0	0	0	0	0
**Lac**	0	0	0.1	0	0	0	0	0
**Leu**	0	0	0.2	0	0	0	0	0
**Lys**	2	2	0	0	0	0	2	2
**Met**	0	0	-0.2	0	0	0	0	0
**NH4**	0	0	0	0	0	0	0	0
**Phe**	0	0	0	0	0	0	0	0
**Pro**	0	0	0.2	0	0	0	0	0
**Ser**	0	0	0.1	0	0	0	0	0
**Thr**	0	0	0.1	0	0	0	0	0
**Trp**	0	0	0	0	0	0	0	0
**Tyr**	0	0	0	0	0	0	0	0
**Val**	0	0	0.2	0	0	0	0	0

Supplementary Figures

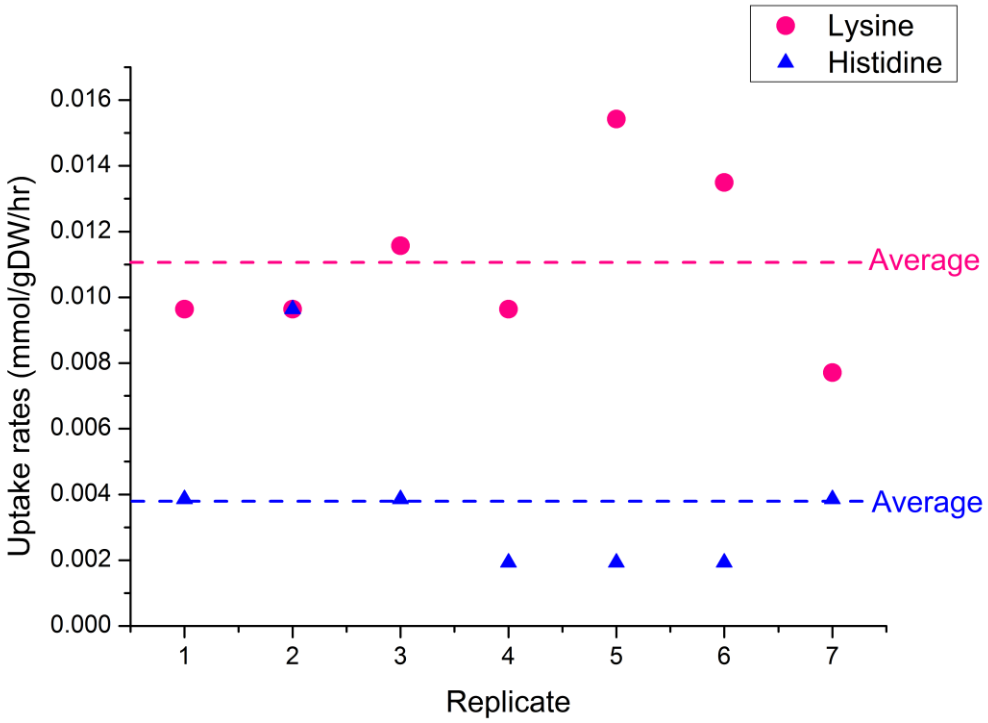

Supplementary Figure 1: Reported uptake rates measurements of lysine and histidine for 7 replicates[19] used as constraints in Figure 1b. Unit converted by assuming cell dry weight = 216.1 pg/cell.

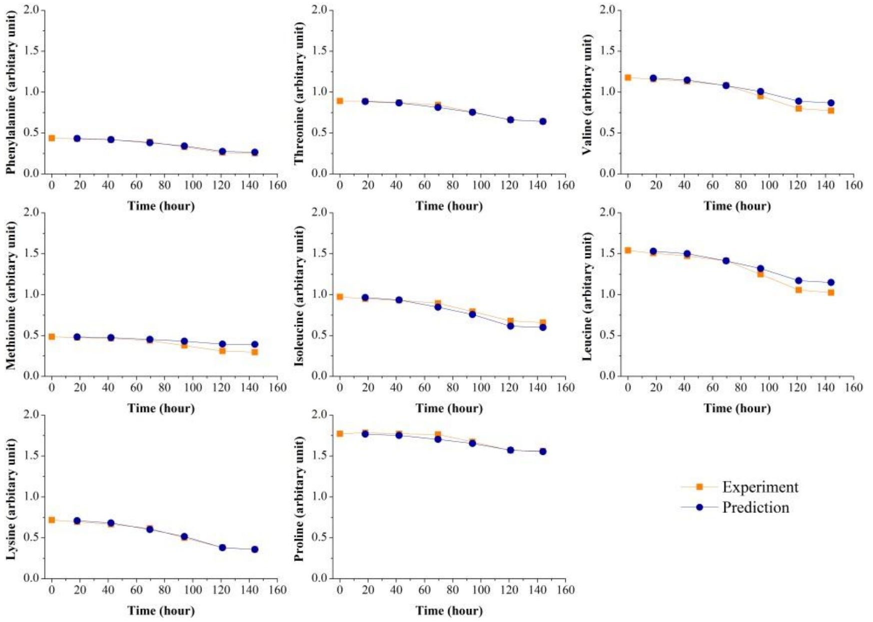

Supplementary Figure 2: Dynamic essential amino acid level estimation of CHO cell batch culture experiment with 100% media A. Prediction results calculated from VCD and initial amino acid levels. Concentration values were normalized in order to protect proprietary media compositions. Simulation results for other cases are attached and reported in values.

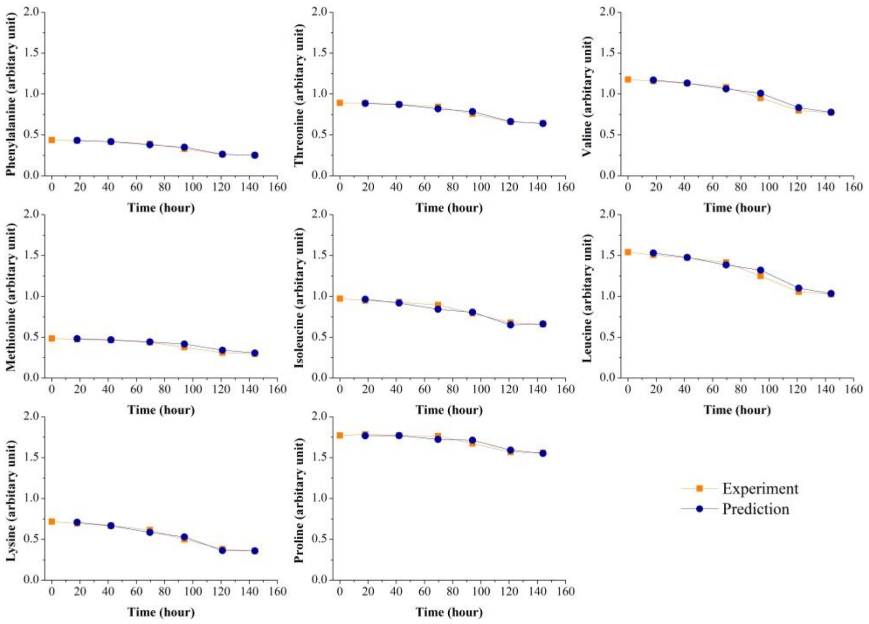

Supplementary Figure 3: Dynamic essential amino acid level estimation of CHO cell batch culture experiment with 100% media A, with off-line data correction. Prediction results calculated from VCD and off-line amino acid measurements at previous time points. Concentration values were normalized in order to protect proprietary media compositions. Simulation results for other cases are attached and reported in values.

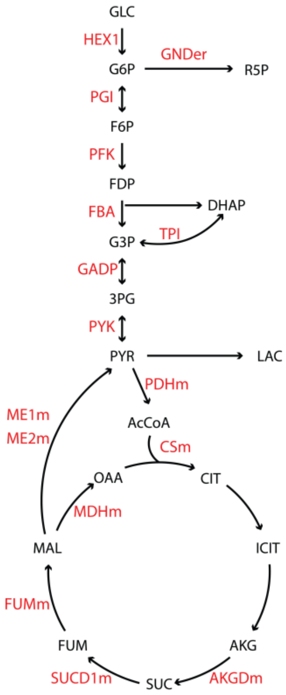

BOF

Reaction	K1 Min	K1 Max	DG44 Min	DG44 Max	S Min	S Max
HEX1	0	1.380	0	0.672	0	2.514
PGI	-1.560	0.191	-1.030	0.191	-2.508	0.191
GNDer	0	0.558	-1000	0.558	0	0.558
PFK	-1000	0.186	-1000	0.186	-1000	0.186
FBA	0	0.186	0	0.186	0	0.186
TPI	-0.747	1000	-0.382	0.184	-0.736	1000
GAPD	-0.563	1000			-0.553	1000
PYK	-1000	2.788			-1000	2.756
PDHm	-1000	0.581			-1000	0.302
CSm	-1000	2.609	0	0.731	-1000	2.577
AKGDm	0	0.375			0	0.375
SUCD1m	-0.534	0.382	-0.004	0.439	-0.100	0.382
FUMm	0	1000	-1000	1000	-1000	1000
MDHm	0	1000	-0.413	0.908	-1000	1000
ME1m	-1000	2.427	-1000	0.520	-1000	2.395
ME2m	0	2.427	0	0.520	0	2.395

UOFs (glucose uptake solution)

Reaction	K1 Min	K1 Max	DG44 Min	DG44 Max	S Min	S Max
HEX1	0	0.171	0	0.196	0	0.172
PGI	-0.007	0.164	-0.549	0.189	-0.007	0.164
GNDer	0	0.000	-1000	0.541	0	0.000
PFK	-1000	0.158	-1000	0.180	-1000	0.159
FBA	0	0.158	0	0.180	0	0.159
TPI	0.156	1000	-0.207	0.178	0.156	1000
GAPD	0.312	1000			0.313	1000
PYK	-1000	0.382			-1000	0.383
PDHm	-1000	0.237			-1000	0.238
CSm	-1000	0.171	0	0.220	-1000	0.172
AKGDm	0	0.209			0	0.209
SUCD1m	0.210	0.213	0.085	0.356	0.210	0.214
FUMm	-1000	1000	-1000	1000	-1000	1000
MDHm	-1000	1000	0.040	0.388	-1000	1000
ME1m	-1000	0.073	-1000	0.044	-1000	0.074
ME2m	0	0.073	0	0.044	0	0.074

Supplementary Figure 4: Flux variability profiles of selected reactions for early exponential case in Figure 4b and 4c, solved by BOF and UOFs (glucose uptake minimization). Minimal and maximal viable flux ranges of reactions were solved by flux variability analysis.

Supplementary References

1 Ahn, W. S. & Antoniewicz, M. R. Parallel labeling experiments with [1,2-13C]glucose and [U-13C]glutamine provide new insights into CHO cell metabolism. *Metab. Eng.* **15,** 34–47 (2013).

2 Ahn, W. S. & Antoniewicz, M. R. Metabolic flux analysis of CHO cells at growth and non-growth phases using isotopic tracers and mass spectrometry. *Metab. Eng.* **13,** 598–609 (2011).
